# Diagnostic value of biochemical ratios in the differential diagnosis of tuberculous and malignant pleural effusions

**DOI:** 10.1186/s12890-026-04421-w

**Published:** 2026-07-03

**Authors:** Umut İlhan, Zeynep Güney, Ayşe Özçelik, Helin Su Kaya, Erdogan Cetinkaya, Mustafa Çörtük

**Affiliations:** Department of Chest Diseases, University of Health Sciences Turkey, Yedikule Chest Diseases and Thoracic Surgery Training and Research Hospital, Istanbul, Turkey

**Keywords:** Pleural Effusion, Tuberculosis, Pleural Neoplasms, Adenosine Deaminase, Lactate Dehydrogenase

## Abstract

**Background and objective:**

The differential diagnosis between tuberculous pleural effusion (TPE) and malignant pleural effusion (MPE) in patients with pleural effusion (PE) continues to pose significant clinical challenges. While conventional pleural fluid cytology has limited sensitivity, invasive diagnostic procedures are often costly or inaccessible, particularly in resource-limited settings. In this study, we investigated the diagnostic contribution of ratios derived from pleural fluid adenosine deaminase (ADA), total protein, serum lactate dehydrogenase (S-LDH), and pleural effusion lactate dehydrogenase (PE-LDH) levels in patients with histopathological confirmed pleural effusion etiology. Specifically, the diagnostic performance of biochemical and hematological parameters, particularly the ADA/serum protein, S-LDH/ADA and PE-LDH/ADA ratios were evaluated for their ability to discriminate between TPE and MPE.

**Methods:**

This retrospective cross-sectional study was conducted among patients who underwent diagnostic evaluation and treatment for pleural effusion at a tertiary referral hospital between 1 November 2014 and 1 November 2024. A total of 1,003 patients with pleural effusion (397 TPE and 606 MPE: 409 primary, 133 metastatic, and 64 mesothelioma) were included, whose etiologies were determined using a composite reference standard based on clinical, radiological, microbiological, and histopathological findings, with multidisciplinary team (MDT) evaluation applied in selected cases where appropriate. Receiver operating characteristic (ROC) curve analyses were performed to determine diagnostic accuracy, sensitivity, specificity, and optimal cut-off values.

**Results:**

Patients with TPE were significantly younger than those with MPE (median age: 32 vs. 65 years, *p* < .001). Overall, 62.9% of the study population were male. ADA levels were significantly higher in the TPE group, whereas S-LDH levels were higher in the malignant effusion group. When ratio-based parameters were evaluated, the S-LDH/ADA ratio was markedly higher in patients with malignant pleural effusion and emerged as the most powerful discriminatory marker, with an area under the curve (AUC) of 0.938, sensitivity of 89.5%, and specificity of 91.3%. The ADA/serum protein ratio also demonstrated high diagnostic accuracy for differentiating TPE, with an AUC of 0.929, sensitivity of 89.9%, and specificity of 93.3%. In multivariate analysis, the S-LDH/ADA ratio, ADA/serum protein ratio, and PE-LDH/ADA ratio remained independently associated with diagnostic discrimination between TPE and MPE.

**Conclusions:**

Biochemical ratios particularly the S-LDH/ADA and PE-ADA/serum protein ratios are reliable, minimally invasive diagnostic tools for distinguishing TPE from MPE. These ratios are especially useful in guiding clinical decision-making in settings where access to invasive diagnostic procedures is limited.

**Supplementary Information:**

The online version contains supplementary material available at https://doi.org/10.1186/s12890-026-04421-w.

## Introduction

Fluid accumulation within the pleural space typically develops as a result of a pathological disruption of the normal balance between hydrostatic and oncotic pressures in the pleural capillaries and lymphatic drainage [1]. PE is classified as transudative or exudative according to Light’s criteria [2].

Transudative pleural effusions typically result from systemic disturbances in fluid balance such as congestive heart failure, nephrotic syndrome, and liver cirrhosis in which the pleural membranes remain structurally intact. In contrast, exudative pleural effusions arise from underlying pathological conditions most commonly tuberculosis (TB), bacterial infections leading to parapneumonic effusion or empyema and malignancy-related pleural effusion accumulation [3].

The most common causes of exudative pleural effusions include parapneumonic effusion, empyema, malignant pleural effusions, tuberculous pleural effusion, pulmonary embolism, viral infections, and rheumatologic diseases [3]. In developing countries, where tuberculosis remains prevalent and cancer incidence is steadily increasing, establishing a reliable differential diagnosis between TPE and the various subtypes of MPE (including primary tumors, metastatic disease and mesothelioma) is of paramount importance. This distinction directly influences clinical management, therapeutic strategies, and overall prognosis [4].

TPE occurs in approximately 5% of patients with tuberculosis infection whereas MPE represents a clinically important condition that develops in about 15% of all patients with cancer [5]. While TPE generally reflects a potentially curable disease with a favorable response to treatment, MPE is most often associated with advanced-stage malignancy and carries a poorer prognosis [6]. Although radiological assessment and biochemical analysis of pleural effusion are useful for diagnosis, the presence of overlapping clinical symptoms—such as dyspnea, anorexia, and weight loss—in both TPE and MPE may complicate the interpretation of these findings [7].

ADA, an enzyme released by lymphocytes, neutrophils and erythrocytes ; plays a role in the catabolism of purine nucleosides [8]. Elevated ADA levels have been associated with the immune response against intracellular pathogens such as *Mycobacterium tuberculosis*. Pleural ADA is used as a biomarker for the diagnosis of TPE, and the widely accepted diagnostic threshold is 40 U/L [9]. Although early studies reported high sensitivity and specificity for ADA (92% and 90%, respectively), more recent data indicate that despite consistently high sensitivity (88–90%), specificity may be substantially lower (31–78%). ADA can also be increased in other granulomatous diseases such as sarcoidosis, as well as in lymphoma, rheumatologic diseases, parapneumonic effusions, empyema, and certain viral infections. Therefore, elevated ADA levels reduce test specificity, particularly in populations with a low prevalence of tuberculosis [10, 11].

LDH is a ubiquitous intracellular enzyme found in multiple tissues, including the heart, liver, erythrocytes, skeletal muscle and kidneys, and is released into body fluids following cellular injury or lysis. Owing to this characteristic, LDH is widely used as a biomarker across a range of pathological conditions [12]. Measurement of pleural effusion LDH constitutes a core component of Light’s criteria for distinguishing exudative from transudative pleural effusions; pleural LDH levels below two-thirds of the upper limit of normal serum LDH are suggestive of a transudative effusion, whereas higher levels indicate an exudative process [13]. However, because LDH levels may be similarly elevated in both tuberculous and malignant pleural effusions, reliance on LDH alone may result in diagnostic misclassification. Consequently, the combined use of LDH with other biomarkers and derived ratios is considered a more reliable diagnostic approach [13, 14].

Pleural effusion cytological examination is a primary diagnostic method for MPE; however, its sensitivity is limited (~ 60%) particularly in mesothelioma and non-shedding tumors [15]. Although invasive diagnostic procedures such as thoracoscopic biopsy provide high accuracy for histological confirmation, they are invasive, costly, and may be difficult to access in resource-limited settings [16].

Given these challenges, there has been increasing interest in diagnostic ratios derived from pleural and serum biochemical markers (e.g., ADA, LDH, glucose, and protein) as well as systemic inflammation indices such as the neutrophil-to-lymphocyte ratio (NLR) [17, 18]. In this study, we aimed to evaluate the diagnostic value of various biochemical and hematological ratios in differentiating TPE from MPE, including its subtypes (primary, metastatic, and mesothelioma).

## Materials and methods

This retrospective cross-sectional study was conducted between November 1, 2023, and November 1, 2024. Patient data were retrospectively collected from medical records covering the period between November 1, 2014, and November 1, 2024. Ethical approval was obtained from the institutional ethics committee (Decision No.: 2023 − 416).

**Fig. 1:**
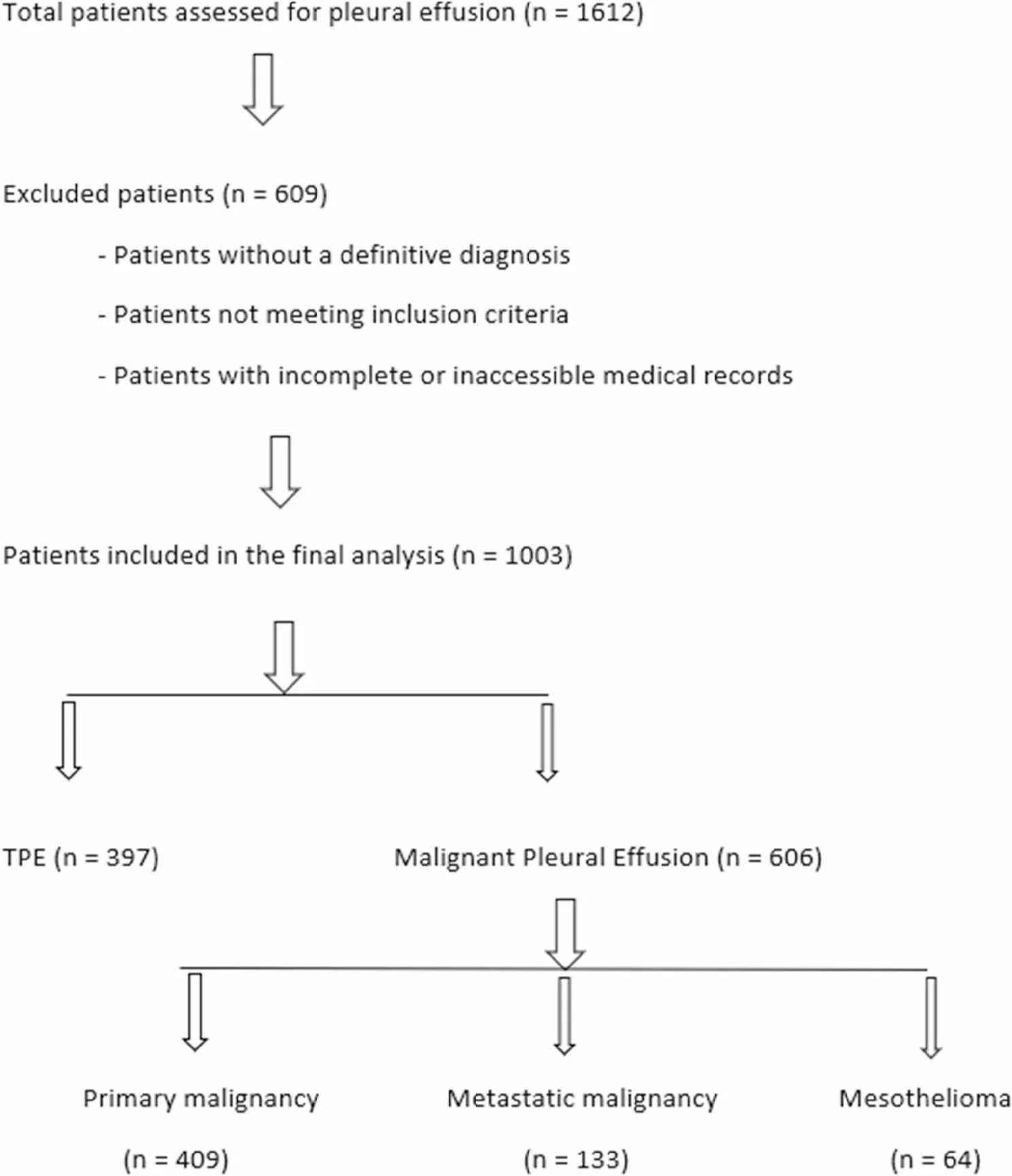
Flowchart of patient selection, exclusion process

A total of 1612 patients with pleural effusion were initially assessed. Of these, 609 patients were excluded as they did not meet the inclusion criteria, including those with pleural effusions other than TPE and MPE (such as transudative effusions, parapneumonic effusions, and empyema). The patient selection process is illustrated in the flowchart (Fig. 1).

### Patient selection criteria

The inclusion criteria were as follows: individuals aged ≥ 18 years of either sex or patients with pleural effusion confirmed by imaging modalities. Patients whose pleural effusion etiology was established using a composite approach based on the combined evaluation of clinical, radiological, microbiological, and histopathological findings were included in the study, with multidisciplinary team (MDT) evaluation performed in selected cases when required.

The exclusion criteria were as follows: age < 18 years, an undetermined etiology of pleural effusion, or incomplete or inaccessible medical records.

The data was collected using a structured clinical data form specifically developed for this study in Microsoft Excel. The form included clinical history, diagnostic findings and the etiological classification of pleural effusion.

The diagnosis of TPE was established based on the presence of at least one of the following criteria: pleural fluid ADA level ≥ 40 U/L in conjunction with compatible clinical and radiological findings; demonstration of granulomatous inflammation in pleural fluid or pleural biopsy; identification of caseating granulomas on pleural biopsy; or detection of *Mycobacterium tuberculosis* in pleural fluid by culture or PCR.

Pleural effusion ADA ≥ 40 U/L was not used as a standalone inclusion criterion but was interpreted in conjunction with clinical and radiological findings as part of routine diagnostic practice.

Patients were categorized into two main groups: TPE and malignancy. The malignancy group was further subdivided into three subgroups: primary malignancy, metastatic malignancy and mesothelioma.

### Measurement of biochemical parameters

All biochemical parameters were analyzed in the central laboratory using a fully automated analyzer (Mindray BS-800 M, Mindray Bio-Medical Electronics Co., Shenzhen, China). Pleural effusion samples were centrifuged prior to analysis to remove cellular components. Measurements were performed using spectrophotometric methods. ADA levels were determined using a kinetic enzymatic colorimetric method based on the Giusti and Galanti principle. S-LDH and PE-LDH levels were measured using an enzymatic rate method (UV kinetic assay), and total protein levels were determined using the biuret colorimetric method. All analyses were performed in accordance with the manufacturer’s instructions and standard laboratory quality control procedures.

### Statistical analysis

Data were analyzed using the Jamovi-R statistical software package (version 2.3.28) [19–24]. Descriptive statistics were presented as counts (n), percentages (%), median, and interquartile range (IQR). The normality of the distribution of continuous variables was assessed using the Shapiro–Wilk test. For comparisons of continuous variables between two groups, the independent samples *t*-test was applied when data were normally distributed, whereas the Mann–Whitney U test was used when normality assumptions were not met.

For comparisons of continuous variables among more than two groups, one-way analysis of variance (Welch’s ANOVA) and the Kruskal–Wallis test was used, as appropriate. Comparisons of categorical variables between groups were performed using chi-square analyses (Pearson’s chi-square, Yates’ continuity-corrected chi-square, and Fisher’s exact test). When chi-square analyses yielded statistically significant results, subgroup analyses were conducted using the two-proportion Z test with Bonferroni correction. For each patient, the following diagnostic ratios were calculated: ADA/serum protein ratio, S- LDH/ADA ratio, and PE- LDH/ADA ratio. ROC curve analyses were performed to assess the discriminative power of these ratios in differentiating TPE from MPE. Optimal cutoff values were determined based on the Youden index, and sensitivity, specificity, positive predictive value (PPV), and negative predictive value (NPV) were calculated.

To evaluate the independent diagnostic value of the biomarkers and the ratios derived from these biomarkers, univariate and multivariate binary logistic regression analyses were performed, with tuberculous pleural effusion defined as the target outcome [1] and primary malignancy as the reference group (0). In the univariate logistic regression analysis, age, ADA level, S-LDH, serum protein, and all derived ratios were included in the model. In the multivariate logistic regression analysis, these parameters were evaluated simultaneously to investigate whether the derived ratios provided diagnostic information independent of their constituent biochemical parameters. Regression results were presented as odds ratios (ORs) with 95% confidence intervals.

To evaluate the stability of the multivariable binary logistic regression model and ensure it was not prone to increased variance from multicollinearity, collinearity diagnostics were performed. The variance inflation factor (VIF) for all included predictors was 1.89, confirming excellent model stability and the absence of significant multicollinearity.

The area under the ROC AUC was used to assess overall diagnostic performance. All statistical tests were two-tailed, and a *p* value < 0.05 was considered statistically significant.

To validate the internal stability and safeguard against potential threshold optimism or sample-dependent overfitting of the clinically proposed operational cut-off points, an automated non-parametric bootstrapping procedure was executed. The original clinical dataset was randomly resampled with replacement across 1,000 independent iterations. For each bootstrap pseudo-dataset, the optimal diagnostic cut-off threshold was independently recalculated by maximizing the Youden index. The distribution of these simulated thresholds was used to derive the bootstrap median, standard error (SE), bias estimate, and 95% percentile confidence intervals (95%CI) for each biomarker ratio.

## Results

### Baseline demographic and clinical characteristics: tuberculosis vs. malignancy

A total of 1,003 patients were included in the study; 397 were diagnosed with TPE and 606 with malignant pleural effusion (MPE) (Fig. 1). Among patients with malignant pleural effusion (*n* = 606), pleural fluid cytology was diagnostic in 144 cases (23.8%). Histopathological confirmation was obtained via pleural biopsy in 213 patients (35.1%) and via VATS in 189 patients (31.2%). Additionally, 112 patients (18.5%) were diagnosed based on multidisciplinary team (MDT) consensus integrating clinical, radiological, and laboratory findings. Among cytology-positive cases, adenocarcinoma was the most common subtype. The median age was significantly higher in the malignancy group than in the TPE group (65 years [IQR: 17] vs. 32 years [IQR: 22], *p* < .001). Male patients predominated in both groups (TPE: *n* = 244 [61.4%]; MPE: *n* = 387 [63.8%]); however, this difference was not statistically significant (*p* = .442). Table 1 summarizes the laboratory parameters and baseline demographic characteristics.

ADA levels and PE-LDH levels were significantly higher in patients with TPE than in those with malignancy (*p* < .001). In contrast, S- LDH levels were significantly higher in patients with malignancy compared with those with TPE (*p* < .001).

With respect to hematological parameters, patients with malignancy had higher white blood cell (WBC) counts (*p* < .001). However, no statistically significant difference was observed between the two groups in terms of the NLR (*p* = .214).

When diagnostic ratios incorporating multiple parameters were evaluated, the ADA/serum protein ratio was significantly higher in patients with TPE than in those with malignancy (0.615 [IQR: 0.34] vs. 0.128 [IQR: 0.103], *p* < .001). In contrast, the S-LDH/ADA ratio (29.857 [IQR: 30.027] vs. 4.828 [IQR: 3.291], *p* < .001) and the PE-LDH/ADA ratio (42.667 [IQR: 50.810] vs. 11.372 [IQR: 8.614], *p* < .001) were significantly higher in the malignancy group.

The details of single and multiparameter analyses are presented in Table 1.

**Table 1:** The comparison of demographic, clinical, biochemical, and hematologic parameters between patients

Variable	Malignancy (*N* = 606)	TPE(*N* = 397)	Test Statistic
Age	65 (17)	32 (22)	*p*<.0011
Sex			
Male	387 (63.8%)	244 (61.4%)	0.4422
Female	219 (36.2%)	153 (38.6%)	
Pleural Effusion			
Right	366 (60.8%)	217 (55.1%)	0.0032
Left	203 (33.7%)	168 (42.6%)	
Bilateral	33 (5.5%)	9 (2.3%)	
ADA	8.8 (7.3)	45 (25)	*p*<.0011
PE Glucose	109 (47)	83 (24.5)	*p*<.0011
PE-LDH	360 (418)	517 (381.25)	*p*<.0011
PE Ph	7.48 (0.17)	7.44 (0.133)	*p*<.0011
PE Protein	45 (10.6)	53 (7)	*p*<.0011
PE Albumin	26.9 (8)	29 (5)	*p*<.0011
Serum Glucose	118 (48)	102 (25)	*p*<.0011
Serum Protein	68 (11)	74 (9.95)	*p*<.0011
Serum Albumin	37 (8)	37 (7)	0.0941
Serum LDH	250 (142.75)	218 (73)	*p*<.0011
WBC	8830 (4465)	7720 (2602)	*p*<.0011
Neutrophil Count	6535 (3742)	5285 (2407)	*p*<.0011
Lymphocyte Count	1520 (1000)	1390 (730)	*p*<.0011
Neutrophil/lymphocyte ratio	3.959 (4.404)	3.929 (3.048)	0.2141
PE ADA/ Serum Protein	0.128 (0.103)	0.615 (0.34)	*p*<.0011
Serum LDH/PE ADA ratio	29.857 (30.027)	4.828 (3.291)	*p*<.0011
PE LDH/PE ADA ratio	42.667 (50.810)	11.372 (8.614)	*p*<.001

Abbreviation: N is the number of patients. 1 Mann–Whitney U test. 2 Chi-squared test (Pearson) *PE* Pleural Effusion, *ADA* Adenosine Deaminase, *LDH* Lactate Dehydrogenase, *WBC* White Blood Cell

### Comparison of cancer subtypes (primary vs. metastatic vs. mesothelioma)

Significant differences were observed among the three groups—primary lung malignancy (*n* = 409), metastatic malignancy (*n* = 133), and mesothelioma (*n* = 64) and the findings are presented in Table 2.

Age and sex distribution differed significantly among the three groups (*p* = .0041 and *p* = .0052, respectively). The proportion of male patients was highest in the mesothelioma group (67.2%), and these patients were of more advanced age. Compared with the primary and metastatic groups, ADA levels were significantly higher in the mesothelioma group (median: 12 U/L, *p* < .001). Similarly, pleural glucose levels (*p* < .001) and pleural pH (*p* = .0031) were significantly lower in patients with mesothelioma.

The NLR was significantly higher in the primary malignancy group (*p* < .001). The highest ADA/serum protein ratio was observed in patients with mesothelioma (median: 0.176); however, the S-LDH/ADA and PE-LDH/ADA ratios in this group were significantly lower (both *p* < .001).

**Table 2:** Presents a comparison of demographic, clinical, and biochemical characteristics across four diagnostic groups: tuberculosis, primary malignancy, metastatic carcinoma, and mesothelioma

	Primary Malignancy (*N* = 409)	Metastatic (*N* = 133)	Mesothelioma (*N* = 64)	Test Statistic
Age	66	62	62	0.004^1^
Sex				0.005^2^
Male	275	69	43	
Female	134	64	21	
Pleural Effusion				0.046^2^
Right	253	74	39	
Left	140	44	19	
Bilateral	15	14	4	
PE-ADA	8	9.5	12	*p*<.001^1^
PE Glucose	110	112	83.5	*p*<.001^1^
PE LDH	388.5	341	338.5	0.676^1^
PE Ph	7.49	7.5	7.44	0.003^1^
PE Protein	44	45	47	0.007^1^
PE Albumin	26	27	28	0.015^1^
Serum Glucose	118	121	109	0.076^1^
Serum Protein	68	69.5	72.5	0.002^1^
Serum Albumin	36	38	39	*p*<.001^1^
Serum LDH	259	251	186.5	*p*<.001^1^
WBC	9775	7190	7940	*p*<.001^1^
Neutrophil Count	7020	5650	5769.4	*p*<.001^1^
Lymphocyte Count	1480	1520	1685	0.075^1^
Neutrophil/lymphocyte ratio	4.602	3.173	3.497	*p*<.001^1^
ADA/ Serum Protein	0.119	0.145	0.176	*p*<.001^1^
Serum LDH/ADA ratio	33.863	26.477	16.206 (IQR13.7656)	*p*<.001^1^
PE LDH/ADA ratio	47.583 (IQR59.7974)	37.250 (IQR 48.4248)	27.598 (IQR 23.2075)	*p*<.001^1^

Abbreviation: N is the number of patients. ^1^Kruskal-Wallis. ^2^Chi-squared test (Pearson) *PE* Pleural Effusion, *ADA* Adenosine Deaminase, *LDH* Lactate Dehydrogenase, *WBC* White Blood Cell

### Receiver Operating Characteristic (ROC) curve analysis

**Fig. 2:**
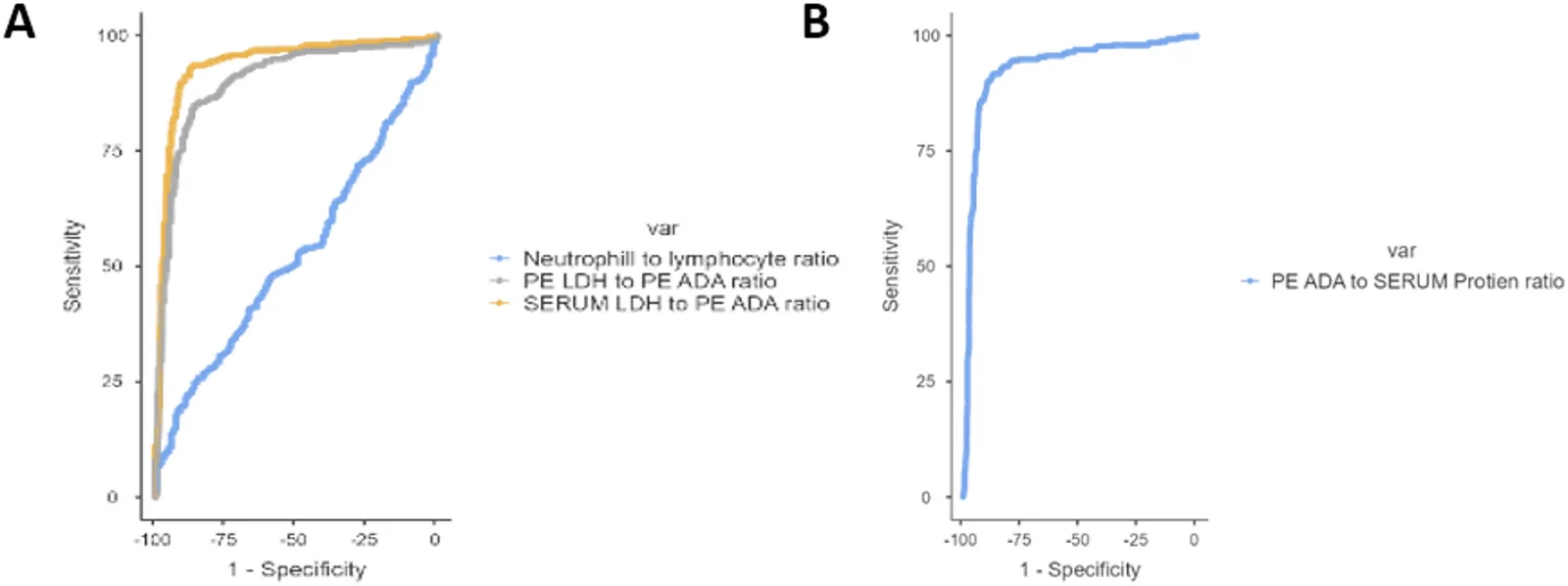
Receiver Operating Characteristic (ROC) curve analysis for differentiating tuberculous pleural effusion (TPE) from primary malignant effusion. **A** Neutrophil-to-lymphocyte ratio (NLR). **B** ADA/serum protein ratio

ROC curve analyses were performed to differentiate TPE from MPE. The NLR was higher across all malignancy groups but demonstrated limited diagnostic value (AUC: 0.577; sensitivity: 31.1%; specificity: 84.7%) (Fig. 2A). In contrast, the S-LDH/ADA ratio exhibited the highest discriminative power, with an AUC of 0.938 (Youden index = 0.808). At a cutoff value of 11.14, sensitivity and specificity were 89.5% and 91.3%, respectively. Similarly, for all malignancies, the PE-LDH/ADA ratio showed high diagnostic performance at a cutoff value of 20.75, yielding an AUC of 0.901, sensitivity of 84.8%, and specificity of 86.3%.

The ADA/serum protein ratio demonstrated high discriminative ability for distinguishing tuberculous pleural effusions. In ROC analysis, the AUC was 0.929. At the optimal cutoff value of 0.305, sensitivity and specificity were 89.95% and 89.5%, respectively. PPV was 84.71%, and the negative NPV was 93.23% (Fig. 2B).

The S-LDH/ADA ratio emerged as the most reliable marker for differentiating primary malignancy from tuberculous pleural effusion, with an AUC of 0.956. This ratio demonstrated high sensitivity (94.58%) and specificity (91.28%) for identifying malignancy and excluding tuberculosis. Similarly, the PE-LDH/ADA ratio also provided high diagnostic utility, with an AUC of 0.924, sensitivity of 94.58%, and specificity of 91.28% (Table 3).

**Table 3:** Diagnostic performance of biomarker ratios for differentiating tuberculous and malignant pleural effusions and their subtypes

Scale/Ratio	Cut point	Sensitivity (%) (95% CI)	Specificity (%) (95% CI)	PPV (%) (95% CI)	NPV (%) (95% CI)	LR+ (95% CI)	LR- (95% CI)	AUC (95% CI)	Youden’s index
Neutrophil/Lymphocyte ratio	6.98	31.10% (26.43–36.07)	84.72% (80.58–88.28)	67.84% (61.30–73.74)	54.27% (52.25–56.27)	2.04 (1.53–2.71)	0.81 (0.75–0.88)	0.577 (0.535–0.618)	0.158
S-LDH/ ADA ratio	11.22	94.58% (91.91–96.57)	91.28% (88.03–93.89)	91.87% (89.11–93.97)	94.18% (91.50–96.05)	10.85 (7.86–14.97)	0.06 (0.04–0.09)	0.956 (0.941–0.972)	0.859
PE-LDH/ADA ratio	20.75	93.10% (90.19–95.37)	90.21% (86.81–92.98)	90.87% (88.02–93.09)	92.59% (89.72–94.71)	9.51 (7.02–12.87)	0.08 (0.05–0.11)	0.946 (0.928–0.963)	0.76
ADA/Serum Protein ratio	0.3049	89.22% (85.79–92.05)	87.02% (83.29–90.18)	87.71% (84.65–90.23)	88.60% (85.43–91.15)	6.88 (5.31–8.90)	0.12 (0.09–0.16)	0.924 (0.905–0.944)	0.833

Abbreviation: *ROC* Receiver Operating Characteristic, *AUC* Area Under the Curve, *CI* Confidence Interval, *PPV* Positive Predictive Value, *NPV* Negative Predictive Value, *Sens* Sensitivity, *Spec* Specificity

Internal validation via 1,000 bootstrap resamples confirmed the structural stability and reproducibility of the mathematically optimized diagnostic thresholds derived from the primary cohort (Supplementary Table 1). The original cohort cut-off point for the premier independent index, the S-LDH/PE-ADA ratio (11.22), demonstrated close alignment with the bootstrap median threshold (10.95), yielding a stable 95% percentile confidence interval of 9.61 to 12.67 (SE = 0.66; Bias = -0.32). Similarly, high threshold stability was established for the PE-ADA/serum protein ratio, where the original sample cut-off of 0.30 fell securely within a tight bootstrapped interval of 0.27 to 0.37 (Median = 0.29; SE = 0.02). The peak operational cut-off point for the PE-LDH/PE-ADA ratio (20.75) displayed identical consistency with the bootstrap median threshold (21.35; 95% CI: 20.73 to 26.16). Conversely, the conventional neutrophil-to-lymphocyte ratio (6.98) exhibited a wider threshold distribution (Median = 6.74, 95% CI: 4.22 to 8.78; SE = 1.37), underling its limited comparative diagnostic reliability. These simulation parameters confirm that the calculated biochemical ratio thresholds are mathematically stable and highly generalizable.

Furthermore, to comprehensively evaluate the predictive accuracy of the individual continuous parameters across their entire measurement distributions, univariate calibration architectures were evaluated using non-parametric locally estimated scatterplot smoothing (LOESS) curves (Supplementary Figs. 1–4). The premier diagnostic metric, the Serum LDH / PE-ADA ratio, demonstrated exceptional calibration accuracy, with its tracking line adhering tightly to the ideal 45-degree reference standard and exhibiting minimal optimism across all probability spectra. High-precision, stable calibration paths were similarly observed for both the PE-ADA / Serum Protein ratio and the PE-LDH / PE-ADA ratio, confirming a highly reliable and consistent relationship between their predicted risk probabilities and actual observed tuberculous etiologies. In stark contrast, the univariate calibration curve for the conventional systemic inflammation index, the Neutrophil-to-Lymphocyte Ratio (NLR), displayed profound structural deviations from the ideal reference line accompanied by significantly widened 95% confidence boundaries. This visual and statistical calibration instability underscores the limited clinical utility of standalone NLR for targeted etiologic discrimination, while reinforcing the robustness and reliability of the novel biochemical ratio frameworks.”

To identify independent predictors of TPE compared with MPE, univariate and multivariate logistic regression analyses were performed (Table 4). After adjustment for potential confounders in the multivariate model, age, the S-LDH/ ADA ratio, the ADA/serum protein ratio, and the PE-LDH/pleural ADA ratio were identified as significant independent predictors. Notably, an increase in the ADA/serum protein ratio was strongly associated with a higher likelihood of tuberculosis (adjusted OR: 4.579; 95% CI: 1.723–32.408; *P* = .005). In contrast, older age (adjusted OR: 0.919; 95% CI: 0.906–0.932; *P* < .001) and a higher serum LDH/pleural ADA ratio (adjusted OR: 0.937; 95% CI: 0.910–0.963; *P* < .001) were associated with a lower probability of tuberculosis and were interpreted as favoring malignancy. ADA and S-LDH, which were significant in the univariate analysis, lost statistical significance in the multivariate model (*P* = .724 and *P* = .130, respectively).

**Table 4:** Logistic Regression Analysis of Independent Predictors for Primary Malignancy versus Tuberculosis

Variable	Univariate OR (95% CI)	Univariate *P*	Multivariate OR (95% CI)	Multivariate *P*
AGE	0.894 (0.882–0.906)	< 0.001	0.919 (0.906–0.932)	**< 0.001**
ADA	1.093 (1.081–1.105)	0.015	1.021 (0.990–1.053	0.724
S-LDH	0.995 (0.994–0.997)	< 0.001	0.998 (0.995–1.001)	0.130
Serum Protein	1.000 (1.000–1.000)	0.608	1.000 (--1.000)	0.561
S-LDH / ADA Ratio	0.874 (0.857–0.891)	< 0.001	0.937 (0.910–0.963)	**< 0.001**
ADA / Serum Protein Ratio	453.0 (208.0-986.4)	< 0.001	4.579 (1.723–32.408)	**0.005**
PE-LDH /ADA Ratio	0.967 (0.961–0.974)	< 0.001	1.009 (1.005–1.014)	**< 0.001**

Bold values indicate statistically significant results (*p* < 0.05)

Abbreviation: *ADA* Adenosine Deaminase, *LDH* Lactate Dehydrogenase, *PE* Pleural Effusion

## Discussion

This study demonstrates that ratios calculated from routinely available pleural effusion and serum biochemical markers can reliably differentiate between TPE and MPE. Among the indices evaluated, the S-LDH/ADA ratio and the ADA/serum protein ratio showed very high diagnostic accuracy, with both parameters yielding sensitivity and specificity values exceeding 90%. These findings highlight the substantial potential of minimally invasive and low-cost biomarkers, particularly in resource-limited settings or regions with a high disease burden. Given the marked age difference between patients with tuberculosis-related pleural effusion and those with malignancy-related pleural effusion, multivariate analysis was performed, and the S-LDH/ADA ratio, ADA/serum protein ratio, and PE-LDH/ADA ratio were confirmed to remain strong discriminatory markers independent of age.

Accurate differentiation between TPE and MPE remains a major diagnostic challenge, particularly in regions with a high tuberculosis burden and in a world where the age of onset of malignancy is steadily decreasing [23, 24]. A rapid, noninvasive, and cost-effective diagnostic approach is crucial, as conventional methods such as cytology have low sensitivity. Procedures such as thoracoscopy and closed pleural biopsy are invasive, costly, and resource-dependent [25, 26]. In this context, routinely available pleural and serum biochemical tests—such as ADA, LDH, and derived ratios—have attracted increasing interest as practical diagnostic tools [23, 24, 27].

When reviewing studies conducted for differential diagnosis, Aktürk et al. evaluated NLR values in a retrospective study including 158 patients with exudative pleural effusion and reported that the diagnostic performance of this parameter for differential diagnosis was limited [28]. In that study, at low cut-off values (e.g., 0.4), sensitivity for tuberculous pleurisy reached 90%, whereas specificity was markedly low. In contrast, although higher cut-off values (e.g., 8.8 and 9.5) provided better specificity (80–85%), sensitivity decreased substantially, and among patients with TPE and NLR ≥ 9.5, sensitivity remained as low as 8.8%. These findings indicate that pleural NLR generally has low discriminatory power. Similarly, Lee et al., in a retrospective analysis of 104 pleural effusion cases, reported that a pleural effusion NLR cut-off value of 3.0 provided only moderate diagnostic accuracy in differentiating tuberculous pleural effusion from malignant pleural effusion [29]. At this threshold, sensitivity was 66%, specificity was 84%, and the AUC was 0.751 (CI: 0.658–0.844), suggesting that pleural NLR may be useful but is not an optimal biomarker. In our study, NLR also demonstrated low diagnostic accuracy; at a cut-off value of 6.98, the AUC was 0.577, with a sensitivity of 31.1% and a specificity of 84.7%. These results were consistent with previous studies.

In the literature, data evaluating the diagnostic performance of the PE-LDH/ADA ratio in differentiating parapneumonic effusion (PPE) from TPE are limited. In a multicenter retrospective study, Gao et al. evaluated 266 patients with pleural effusion and reported that the PE-LDH/ADA ratio was highly effective in distinguishing PPE from TPE. At a cut-off value of 14.53, the area under the curve (AUC) for the diagnosis of TPE was 0.947, with a sensitivity of 79.4% and a specificity of 100%. In the overall cohort, using a cut-off value of 14.29, the AUC was 0.888, with a sensitivity of 81.1% and a specificity of 83.7%. However, PPE were not included in our study population. Therefore, the PE-LDH/ADA ratio was not evaluated for the differentiation between PPE and TPE in our analysis. This difference in study design should be considered when comparing our findings with those reported in the literature.

Ver Hoai et al. evaluated the S-LDH/ADA ratio for differentiating TPE from MPE in a study including 204 patients. The optimal cut-off value was reported as 20; above this threshold, the sensitivity and specificity for MPE were 85% and 79%, respectively, with an AUC of 0.85 [30]. Verma et al. [18] evaluated 163 patients with exudative pleural effusion and reported that when the S-LDH/ADA ratio was > 20, the sensitivity and specificity for malignancy were 98% and 94%, respectively, with a positive likelihood ratio (PLR) of 32.6 and a negative likelihood ratio of 0.03 [18]. Similarly, in another study by Verma et al., a total of 118 patients were analyzed, including 84 (71.2%) MPE and 34 (28.8%) with TPE. The S-LDH/ADA ratio, referred to as the “cancer ratio” showed a sensitivity of 95% (95% CI: 87–98) and a specificity of 85% (95% CI: 68–94) at a cut-off value of > 20. In addition, the AUC was 0.81, with a PLR of 16 and a negative likelihood ratio 0.13 [31].

In our study, the S-LDH/ADA ratio emerged as the strongest discriminatory biomarker. At a cut-off value of 11.14, the AUC was 0.938, with a sensitivity of 89.5%, a specificity of 91.3%, and a Youden index of 0.808. Compared with the literature, our study similarly demonstrated high diagnostic accuracy; however, this performance was achieved at a lower threshold than that reported in many previous studies. This finding suggests that the S-LDH/ADA ratio is a robust marker for differentiating the etiologies of pleural effusion, while optimal cut-off values may vary according to population characteristics and specimen type. Notably, our cohort was larger than those in prior studies (*n* = 1003), and diagnoses were established predominantly by histopathological confirmation with a small proportion determined through multidisciplinary evaluation. In our study, even lower cut-off values maintained high sensitivity and specificity.

In literature, the PE-DH/ADA ratio has frequently been reported as a highly effective biomarker for differentiating MPE from TPE. Zhao et al. retrospectively evaluated 618 patients with pleural effusion and reported that, for the differentiation between TPE and non-TPE, a PE-LDH/ADA ratio cut-off value of < 23.20 yielded an AUC of 0.946 (95% CI: 0.925–0.966), with a sensitivity of 93.9%, a specificity of 87.0%, and a Youden index of 0.809. In the same study, for distinguishing TPE from PPE, a cut-off value of < 24.32 resulted in an AUC of 0.964 (95% CI: 0.939–0.989), with a sensitivity of 94.6% and a specificity of 94.4%. For the differentiation between MPE and TPE, a cut-off value of < 22.92 for TPE yielded an AUC of 0.926 (95% CI: 0.896–0.956), with a sensitivity of 93.4% and a specificity of 80.0%. The authors concluded that these findings demonstrate the superior diagnostic performance of the PE-LDH/ADA ratio compared with PE-LDH or ADA alone [32].

Beukes et al., in a retrospective study including 228 patients, reported that the PE-LDH/ADA ratio was significantly lower in TPE than in non-TPE pleural effusions and suggested that this ratio may be useful for distinguishing pleural TPE from other causes of pleural effusion. The mean PE-LDH/ADA ratio was lower in patients with pleural TPE than in those with alternative diagnoses (6.2 vs. 34.3, *p* < .001) [10]. In a study by Chun-Yee Yo et al. involving 311 patients, a PE-LDH/ADA ratio of < 14.2 (sensitivity: 74.2%; specificity: 90.4%) supported TPE, whereas a ratio of > 14.5 (sensitivity: 79.9%; specificity: 78.5%) supported PPE. In addition, they reported that a PE-LDH/ADA ratio of > 46.7 (sensitivity: 56.3%; specificity: 78.3%) supported MPE due to primary lung cancers [33].

In our study, the PE-LDH/ADA ratio demonstrated high diagnostic performance. At a cut-off value of 20.75, the AUC was 0.901, with a sensitivity of 84.8–89.5%, a specificity of 86.3%, and a Youden index of 0.760. These findings from our large cohort are consistent with Zhao et al. However, in our study, the cut-off values of the PE-LDH/ADA ratio differed from those reported by Beukes et al. and Chun-Yee Yo et al. This discrepancy may be explained by differences in study design and patient populations. In those studies, comparisons were performed between TPE and non-TPE groups, whereas our study specifically compared TPE with MPE. These methodological differences are likely to influence biomarker distributions and, consequently, the optimal thresholds derived from ROC analyses and confirm that the PE-LDH/ADA ratio is a strong biomarker for distinguishing MPE from TPE.

There are only a limited number of studies in the literature reporting that the ADA/serum total protein ratio demonstrates strong performance for the diagnosis of TPE. Shimoda et al. reported that using an ADA/total protein ratio of < 14 yielded a sensitivity of 79.3% and a positive predictive value (PPV) of 75.4% for TPE, suggesting that ADA/ total protein ratios can provide a meaningful contribution to diagnostic algorithms [34].

In our study, the ADA/serum protein ratio also demonstrated strong diagnostic performance. At a cut-off value of 0.305, higher values favored the diagnosis of TPE. The AUC was 0.929, with a sensitivity of 89–90%, a specificity of 93%, a PPV of 84.7–92.8%, an NPV of 90.7–93.2%, and a Youden index of 0.79. The differences in cut-off values compared to previous studies may be explained by differences in patient populations; for instance, Shimoda et al. analyzed a heterogeneous cohort including 174 patients with tuberculous pleurisy, 215 patients with pleural infections other than tuberculous pleurisy, 360 patients with malignant pleural effusion, and 209 patients with other diseases. Compared with previous reports, our findings confirm that the ADA/serum protein ratio is a strong predictor of TPE.

In the flowchart proposed by Shimoda et al., the ADA/total protein ratio was evaluated in patients with ADA ≥ 40 IU/L and PE-LDH < 825 IU/L [34]. Therefore, the ADA/ total protein < 14 threshold was derived from a different patient population than our ADA/serum protein ratio analysis, which included all exudative cases. In addition, methodological differences such as unit variations (e.g., total protein expressed in mg/dL versus g/L) and the resulting numerical discrepancies may also contribute to differences in reported cut-off values. The substantial numerical differences between the cut-off values are therefore attributable to these methodological differences. Although cut-off values may vary numerically across studies due to methodological heterogeneity, both approaches consistently indicate that normalizing ADA to an appropriate protein component enhances the diagnostic ability to differentiate tuberculous pleurisy.

When biochemical biomarkers used in the differential diagnosis of TPE are considered, analyses relying solely on ADA levels appear to provide limited diagnostic value. Although a cut-off value of 40 U/L for ADA has been reported to yield high specificity (98%), the markedly low sensitivity (59%) indicates that a substantial proportion of TPE cases may be missed. Increasing the threshold to 45 U/L maintains specificity but further reduces sensitivity (49%), underscoring the limited clinical reliability of ADA when used alone. In contrast, using ADA in combination with LDH as a ratio significantly improves diagnostic performance.

The high performance of the S-LDH/ADA ratio and the ADA/serum protein ratio suggests that these indices capture the underlying pathophysiology: ADA reflects the cellular immune response in tuberculosis, whereas LDH and protein levels reflect tissue breakdown and oncotic dynamics in malignancy. The consistency of these ratios across different cancer subtypes (primary, metastatic, and mesothelioma) further indicates that they may serve as reliable decision-support tools across diverse clinical settings.

The major strengths of this study include the large sample size (*n* = 1003) and the inclusion of both TPE and MPE subtypes, which enabled comprehensive subgroup analyses. The standardized statistical approach based on ROC analysis provides robust evidence regarding diagnostic accuracy and clinically applicable cut-off values. Moreover, as the study was conducted in a region with a high tuberculosis prevalence, its findings are likely to be highly generalizable to similar populations.

Although univariate analyses suggested that both the raw biomarkers (S-LDH and ADA) and the derived ratios were significant predictors, multivariable logistic regression provided a critical distinction regarding their independent contributions. Contrary to the concern that the PVR might simply reflect the influence of a dominant component, our multivariable model demonstrated that the derived ratios outperformed the raw biomarkers. Specifically, while S-LDH and ADA were significant in univariate analyses, they lost statistical significance after adjustment (*p* = .130 and *p* = .724, respectively). In contrast, theS-LDH/ ADA ratio remained highly significant (*p* < .001; adjusted OR = 0.937), confirming that the ratio itself is a strong independent discriminator between malignancy and tuberculosis, beyond the information provided by S-LDH alone. This finding suggests that the interaction between systemic tissue breakdown/cellular turnover (LDH) and local inflammation (ADA) provides disease-specific diagnostic information that is not captured by either marker in isolation.

This study has several limitations. One important limitation is the potential for incorporation bias. Although the diagnosis of tuberculous pleural effusion was based on a composite reference standard including clinical, radiological, microbiological, and histopathological findings, pleural ADA (≥ 40 U/L) was also included as part of the diagnostic framework. Since ADA is also a component of the evaluated ratios (e.g., LDH/ADA and ADA/serum protein), this may have introduced a degree of dependency between the reference standard and the index tests, potentially leading to an overestimation of diagnostic performance.

Although ADA was not used as an independent cut-off parameter in the statistical analyses and all biochemical parameters were incorporated into ratio-based calculations, it should be emphasized that ADA levels also contributed to the clinical decision-making process, particularly in patients diagnosed with TPE through multidisciplinary team (MDT) evaluation. Therefore, ADA may have indirectly influenced the reference standard, introducing an additional level of dependency between the reference standard and index tests, which may further increase the risk of incorporation bias. Therefore, the findings should be interpreted with caution.

The relatively wide confidence interval observed for the ADA/serum protein ratio in the multivariate analysis indicates a substantial degree of uncertainty in the estimated effect size and reflects limited precision. Although this may be partly related to the distributional properties of the ratio variable, the wide confidence interval suggests that this finding should be interpreted with caution. Despite this, the parameter remained statistically significant, and multicollinearity analysis (variance inflation factor [VIF] = 1.89) confirmed the stability of the model.

In addition, the relatively small sample size of the mesothelioma subgroup (*n* = 64) may limit the statistical power and reliability of subgroup analyses. However, as subgroup comparisons were not among the primary endpoints of this study, these findings were presented in the Results section and were not extensively interpreted in the Discussion; therefore, comparisons between subgroups should be interpreted with caution.

The retrospective and single-center design of the study may limit the ability to fully exclude potential biases and restrict the generalizability of the findings. In addition, inter-laboratory variability may affect the applicability of the proposed cut-off values. Therefore, our findings should be validated in multicenter, prospective studies.

In conclusion, this study highlights the diagnostic value of simple biochemical ratios in differentiating TPE from MPE. The S-LDH/ADA and ADA/serum protein ratios demonstrated superior performance compared with conventional single-parameter biomarkers and may be considered practical, minimally invasive, and easily applicable diagnostic tools. Incorporation of these ratios into clinical practice has the potential to reduce reliance on invasive diagnostic procedures, accelerate the diagnostic process, and facilitate timely initiation of disease-specific treatments, thereby improving patient outcomes.

## Supplementary Information

Supplementary Material 1.

Supplementary Material 2.

## Data Availability

The datasets generated during and/or analyzed during the current study are available from the corresponding author on reasonable request.

## Abbreviations

ADA: Adenosine deaminase
AUC: Area under the curve
CI: Confidence interval
CTD: Connective tissue disease
IQR: Interquartile range
LDH: Lactate dehydrogenase
MPE: Malignant pleural effusion
NLR: Neutrophil-to-lymphocyte ratio
NPV: Negative predictive value
OR: Odds ratio
PCR: Polymerase chain reaction
PE: Pleural effusion
PE-LDH: Pleural effusion lactate dehydrogenase
PPV: Positive predictive value
ROC: Receiver operating characteristic
S-LDH: Serum lactate dehydrogenase
TB: Tuberculosis
TPE: Tuberculous pleural effusion
TRN: Trial registration number
VATS: Video-assisted thoracoscopic surgery
WBC: White blood cell
