# Supplementary material for: Diagnostic value of biochemical ratios in the differential diagnosis of tuberculous and malignant pleural effusions
Source: BMC Pulm Med. 2026 Jul 3;26:421. doi: 10.1186/s12890-026-04421-w (PMC13599273; doi:10.1186/s12890-026-04421-w)
Supplement: Supplementary file 1 [file 12890_2026_4421_MOESM1_ESM.docx]

**Supplementary Table 1. Internal Validation and Stability Assessment of Operational Cut-Off Points via Bootstrap Resampling (1,000 Repetitions)**

| Diagnostic Biomarker / Ratio | Original Sample Cut-off Point | Bootstrap Median Cut-off Point | Standard Error (SE) | Bootstrap Bias Estimate | Bootstrapped 95% Confidence Interval |
| --- | --- | --- | --- | --- | --- |
| S-LDH / PE-ADA ratio | 11.22 | 10.9524 | 0.6649 | -0.3201 | 9.6080 to 12.6683 |
| PE-ADA / Serum Protein ratio | 0.3049 | 0.294 | 0.0221 | 0.0098 | 0.2667 to 0.3713 |
| PE-LDH / PE-ADA ratio | 20.75 | 21.3515 | 1.2292 | 0.4167 | 20.7315 to 26.1585 |
| NLR | 6.98 | 6.7436 | 1.3673 | -0.533 | 4.2196 to 8.7768 |
