# Supplementary material for: Diagnostic value of biochemical ratios in the differential diagnosis of tuberculous and malignant pleural effusions
Source: BMC Pulm Med. 2026 Jul 3;26:421. doi: 10.1186/s12890-026-04421-w (PMC13599273; doi:10.1186/s12890-026-04421-w)
Supplement: Supplementary file 2 [file 12890_2026_4421_MOESM2_ESM.docx]

**Figure 1.** (A) Non-parametric LOESS (locally estimated scatterplot smoothing) calibration curve mapping the univariate relationship between predicted risk probabilities derived from the Serum LDH / PE-ADA ratio and the actual observed proportion of tuberculous pleural effusions (TPE). The red dashed diagonal line indicates perfect calibration ideal paradigms (x = y). The solid blue line tracks actual performance with shaded grey bands representing the 95% confidence intervals.

**Figure 2.** Non-parametric LOESS calibration curve mapping univariate risk prediction probabilities derived from the PE-ADA / Serum Protein ratio against the actual observed proportion of tuberculous pleural effusions (TPE). The red dashed line denotes the ideal 954595-degree calibration standard, demonstrating strong, near-linear agreement across the higher probability spectrum (95>0.5095). Grey shading outlines the corresponding 95% confidence boundaries.

**Figure 3.** Non-parametric LOESS calibration curve evaluated for the continuous PE-LDH / PE-ADA ratio against the actual observed clinical distribution of tuberculous pleural effusions (TPE). The steep sigmoidal trajectory tracks the raw threshold dynamics of the parameter, with the red dashed diagonal line acting as the perfect calibration reference and grey shading indicating the 95% confidence boundaries.

**Figure 4.** Non-parametric LOESS calibration curve displaying the univariate diagnostic risk mapping of the systemic inflammation index, the Neutrophil-to-Lymphocyte Ratio (NLR). The prominent structural deviations from the ideal red dashed line, accompanied by significantly widened 95% confidence bands (grey shading), illustrate poor individual calibration and highlight its limited clinical utility in distinguishing pleural effusion etiologies.
